# Masked Translation Priming With Concreteness of Cross-Script Cognates in Visual Word Recognition by Chinese Learners of English: An ERP Study

**DOI:** 10.3389/fpsyg.2021.796700

**Published:** 2022-01-07

**Authors:** Shifa Chen, Tingting Fu, Minghui Zhao, Yuqing Zhang, Yule Peng, Lianrui Yang, Xiaolan Gu

**Affiliations:** College of Foreign Languages, Ocean University of China, Qingdao, China

**Keywords:** priming effect of cross-script cognate status, priming effect of concreteness, translation asymmetry, N150, N400

## Abstract

Translation equivalents for cognates in different script systems share the same meaning and phonological similarity but are different orthographically. Event-related potentials were recorded during the visual recognition of cross-script cognates and non-cognates together with concreteness factors while Chinese learners of English performed a lexical decision task with the masked translation priming paradigm in Experiment 1 (forward translation: L1–L2) and Experiment 2 (backward translation: L2–L1). N400 effect was found to be closely related to priming effects of cross-script cognate status and concreteness in Experiment 1; and in Experiment 2, N150 and N400 effects were related to priming effects of cross-script cognate status and concreteness, and greater priming effects of cross-script cognate status in cognates than in non-cognates for abstract words were found in the time window of 100–200 ms. Meanwhile, the asymmetry of translation directions was observed in smaller priming effects in forward translation than in backward translation in the time window of 100–200 ms for abstract cognates, and in larger priming effects in forward translation than in backward translation in the time window of 350–550 ms for each type of words. We discussed the roles of phonological activation and concreteness effects in view of the function of N150 and N400 components as well as the relevant models, mainly the Distributed Feature Model and Bilingual Interactive Activation (BIA+) model.

## Introduction

In the domain of psycholinguistic research on bilingualism, endeavors have been taken to answer the question of whether lexical representations from both languages are simultaneously activated during the processing of the word input. Two competing theories are proposed to account for this issue. The language selective hypothesis assumes that the lexical candidates from the given language are only limited to compete, which corresponds to the view of independent lexicons of two languages, while the language non-selective hypothesis, supporting the view of an integrated lexicon, claims that lexical representations from both languages are accessed simultaneously with respect to bilingual word recognition.

Although the agreement has not been reached on how lexicons of two languages are represented in bilingual memory, much of the previous research has supported a shared conceptual system (see Francis, 2005). Based on this assumption, several models have been proposed to account for the structure of bilingual memory. For instance, the Revised Hierarchical Model (RHM, Kroll and Stewart, 1994) assumes that the meaning of L2 words is accessed through their L1 translation equivalents, and with the improvement of second language proficiency, L2 words can directly be accessed without the assistance of L1, indicating that L1 and L2 shared a common conceptual system. Another model also addressing the issue of L1 and L2 representations is the Distributed Feature Model (DFM, de Groot, 1992; van Hell and de Groot, 1998) which believes that the conceptual representations are distributed in one common conceptual system as meaning elements/nodes are shared by words within the same language and across languages. In other words, translation equivalents from two languages share a certain amount of meaning components, depending on the degree of meaning overlap. In the DFM, concrete words are assumed to have a larger meaning overlap across languages than abstract words since abstract words are more context-dependent and have rather different interpretations in different contexts. In the same vein, cognate words (i.e., translation equivalents with a similar form) have more common conceptual components than non-cognate words. Although the models mentioned above can to some extent explain the empirical data obtained by using different paradigms and experimental tasks, they failed to give a detailed description of the processing of word identification, from the onset of a word to the time when it is accessed. Language retrieval models such as the Bilingual Interactive Activation (BIA+) model (Dijkstra and van Heuven, 2002) are implemented in the studies of bilingual memory to describe the processing of word identification. The BIA+ model is in support of the hypothesis that the bilingual lexicon is integrated and accessed in a non-selective manner. The original BIA (Dijkstra and van Heuven, 1998) model assumes that when a string is presented, activation spreads through four layers of connected nodes, from sub-lexical features to letters, words, and finally the language node. However, the BIA model is only concerned with the recognition of orthographic representations and has limitations in accounting for some empirical results. The BIA+ model, on the other hand, incorporates different levels of representations (i.e., phonological, orthographic, and semantic representations) and predicts a sequential activation of the three levels. Subsequently, other models emerge to deal with the issues related to the processing and representation of mental lexicon, such as the Shared Asymmetrical Model (SAM, Dong et al., 2005), the Sense Model (Finkbeiner et al., 2004), the Modified Hierarchical Model (MHM, Pavlenko, 2009), and DevLex-II model on simulating cross-language semantic priming effects (Zhao and Li, 2013). The fact that so many theoretical models are proposed to account for the processing and representation of mental lexicon leads to many experiments conducted to investigate variables that can modulate the recognition of L1 and L2 words, including word type (cognates and non-cognates), concreteness and translation direction (Ferré et al., 2017).

For the research investigating bilingual word recognition based on the BIA+ model, challenges are presented by cognates that have a certain degree of overlap between two languages in any of the three levels: phonology, orthography, and semantics. In several studies examining whether word type affected the reaction time, more robust priming effects have been observed in cognates than in non-cognates, suggesting a facilitation effect of the cognate status (Duñabeitia et al., 2010; Ferré et al., 2017). The DFM (de Groot, 1992; van Hell and de Groot, 1998) accounts for the cognate facilitation effect by assuming that there is a greater degree of meaning overlap in cognates than in non-cognates, which leads to larger priming effects between their corresponding translation equivalents for cognates than for non-cognates. Generally, when an L2 cognate is learned, it is easy to associate this L2 word to the conceptual representation that has already existed in the memory (van Hell and de Groot, 1998). However, even if cognate translation equivalents that are orthographically and phonologically similar have been selected as critical materials to explore cross-language words recognition, it is still troublesome to disentangle phonological status from orthographic similarity in the translation pairs of alphabetic languages. According to Kim and Davis (2003), due to the orthographic similarity between the same-script languages, there is often a competition between the prime and the target. Since phonological information can be clearly separated from orthographic similarity in the translation pairs of cross-script languages, it is easier to investigate the cognate effects by distinguishing the phonological similarity from orthographic information within the cross-script languages.

A few researchers have investigated cross-script cognate priming effects using words from languages that do not share orthographic identity in behavioral experiments (Zhou et al., 2010; Nakayama et al., 2012, 2013; Ando et al., 2014; Zhang et al., 2018) and event-related potential (ERP) studies (Hoshino et al., 2010; Ando et al., 2015). For example, in a lexical decision task and a naming task, Zhou et al. (2010) observed priming effects of homophones with Chinese–English bilinguals, supporting the non-selective mechanism in phonological representation, which is in line with the BIA+ model as there was no orthographic similarity between Chinese and English. Zhang et al. (2018) investigated Chinese–English cognates and found that there was no advantage for Chinese–English cognates in forward translation whereas only English–Chinese cognates produced facilitation effects in backward translation, which may be attributed to different mappings from orthography to phonology between English and Chinese. Nakayama et al. (2012) examined cognates and phonological similar non-cognates for Japanese–English bilinguals in masked phonological priming paradigm, and found that the priming effects of cognates, but not of phonological similarity, were influenced by target frequency and L2 proficiency. Similar results from ERP experiments also confirmed that phonological priming occurred prior to and independent of the influence of word frequency. Ando et al. (2015) investigated the cross-script phonological activation in Japanese–English bilinguals by recording both the ERP data and response data in a lexical decision task. They found a facilitation effect of Katakana primes to phonologically similar English target words, which indicated that there was a shared store of sublexical phonological representations by both Japanese and English, and the cross-script phonological priming effects were the consequence of the activation of the shared sublexical phonological representations. Therefore, it is important to disentangle the phonological factor from orthographic representation during visual word recognition within cross-script languages by exploring the processing of Chinese loan words and their English equivalents (i.e., Chinese–English cognates, like “幽默-humor”) because they are phonologically and semantically similar, but orthographically different, and can be utilized in the investigation of phonological activation in word recognition. Nevertheless, whether the cognate effects disappear or not on the recognition of the targets based on ERP technology for Chinese loan words and their English equivalents remains unclear.

The factor of concreteness has been well-acknowledged in the previous research, either as an independent variable or as a control variable. It has been suggested that concrete words performed discordantly with abstract words in response latencies and N400 amplitudes in both monolingual and bilingual related studies (Zhang et al., 2006; Tolentino and Tokowicz, 2009; Huang et al., 2010; Barber et al., 2013; Palmer et al., 2013; Ferré et al., 2017). The facilitation effect of concreteness in bilingual word representation has been explained by the DFM (de Groot, 1992; van Hell and de Groot, 1998), which states that concrete words share more semantic components than abstract words. van Hell and de Groot (1998) employed a word association task and found that in both within- and between-language associations, cognates and concrete words were more often associated with their translations relative to non-cognates and abstract words. However, some researchers argued that concrete and abstract words shared equivalent concept overlap across languages in view of the similar priming effects observed in experiments, which discredits the claims of the DFM (Francis and Goldmann, 2011; Chen et al., 2014). One reason for the discrepancy in results may be that concreteness effects could only be observed within a certain range of stimulus-onset asynchronies (SOA). Research by Schoonbaert et al. (2009) examined two SOAs (250 and 100 ms) and their results showed that although the main effect of concreteness did not reach significance in both SOAs, the concrete words but not the abstract words produced a significant priming effect with the 100 ms SOA. Ferré et al. (2017) investigated cognate status and concreteness effects in two SOAs (50 and 100 ms), and found concreteness priming effects only in the longer SOA (100 ms). Thus, the concreteness priming effects are sensitive to SOA duration so that researchers need to carefully consider the factor of SOA when exploring the influence of concreteness, cognate status and their interaction effect on the processing of Chinese–English cross-script cognates.

The robustness of priming effects in the related studies often varies with translation directions. Faster responses were observed when L2 target words were preceded by their L1 translation equivalents (Gollan et al., 1997; Kim and Davis, 2003; Basnight-Brown and Altarriba, 2007), while evidence for L2–L1 priming effect in backward translation was not very consistent, with sometimes null priming effects (Schoonbaert et al., 2009; Dimitropoulou et al., 2011). Chen et al. (2020) found the asymmetrical priming effect between Chinese–English and English–Chinese translation directions with a larger N400 amplitude and a longer N400 latency in Chinese–English translation. The asymmetric effect can be explained by the RHM (Kroll and Stewart, 1994), in which the representations of L1 and L2 are qualitatively different, with L2 words less directly connected to the semantics. In contrast, DFM (de Groot, 1992; van Hell and de Groot, 1998) explains this result in a quantitative way in which L1 words have richer semantic representations than L2 words and thus can activate more features within a shorter time, resulting in stronger priming effects in forward translation (de Groot, 1992). The asymmetry is also predicted by the BIA+ model, which assumes that the speed of activation can be influenced by factors such as subjective frequency, and since L2 words have lower accessibilities than L1 words (L2 words are less frequently or recently used), activation spreads more slowly in L2 access than in L1 access. However, most of the previous studies about the asymmetry of translation directions mainly focused on non-cognate translation equivalents. Cognates with both the semantic and phonological overlap between cross-script languages may shed more light on the studies of translation directions.

In light of the research gaps identified above, previous studies mainly concentrated on languages with the same writing system, and it is difficult to clarify whether the facilitation effect of the cognate status is caused by phonological similarity or orthographic information. In addition, although some related studies have used cross-script languages to distinguish between phonological and orthographic promotion of cognates (Zhang et al., 2018), they do not distinguish between abstract cognates and concrete cognates. With high temporal resolution, ERP technology can provide us a more complete picture by showing the processing of the target words in real time, and has been employed to measure the cross-script phonological activation in Japanese–English bilinguals (Ando et al., 2015). Therefore, the present study is to use Chinese–English cross-script cognates with similar pronunciation and meaning but different orthographic information to examine the roles of phonology as well as concreteness effects with masked translation priming paradigm based on ERP technology in two experiments with different translation directions. It aims to examine whether there are translation priming effects for cross-script cognate status and concreteness in both forward and backward translation directions, whether cross-script phonological similarity and concreteness can elicit greater priming effects for cognates and concrete words than for non-cognates and abstract words in two translation directions, respectively, and whether there exists the translation asymmetry in terms of priming effect magnitudes between the two translation directions.

## Experiment 1: L1–L2 (Chinese–English Forward Translation)

### Methods

Experiment 1 examined the role of phonology as well as concreteness effects for Chinese learners of English with a lexical decision task in the masked translation priming paradigm in the L1–L2 translation direction.

#### Participants

Twenty-five Chinese–English bilinguals (14 females; mean age 20.68, *SD* = 0.79) were recruited from a public university in China to participate in the experiment. They were native Chinese speakers majoring in English and all of them had passed the Test for English Majors-Band 4 (TEM4). No immerse experience to learn English for all the participants and they have been learning English in the classroom environment for 10–12 years. A seven-point Likert scale assessment (1 for “quite poor,” 7 for “highly proficient”) was conducted to evaluate their L1 and L2 proficiency, and their self-reported rating for listening, speaking, reading and writing in L1 (Chinese) were 6.48 (*SD* = 0.65), 6.12 (*SD* = 0.97), 6.16 (*SD* = 0.94), 5.40 (*SD* = 1.12), and in L2 (English) with 4.84 (*SD* = 0.99), 4.68 (*SD* = 1.07), 5.44 (*SD* = 0.92), 4.36 (*SD* = 0.76), respectively. A paired-sample *t*-test showed that there were significant differences between L1 and L2 in listening, speaking, reading and writing [*p*s < 0.001, *Cohen’s d*(s) ≥ 1.044]. Therefore, our participants can be treated as unbalanced Chinese–English bilinguals. All of them had normal or corrected-to-normal vision and were right-handed without neurological disease.

#### Materials

Critical stimuli in Experiment 1 were 40 Chinese–English cognate pairs and 40 Chinese–English non-cognate pairs. All the Chinese–English cognate translation pairs were selected from *A Dictionary of Loan Words and Hybrid Words in Chinese* (Liu et al., 1984). Since there were no already existing common corresponding Chinese–English transliterated pairs, Chinese words were coined based on the pronunciation of the English translation equivalents. To make sure that the Chinese and English cognate pairs were indeed translation equivalents to each other, twenty students in English major who did not participate in the experiment were asked to translate them. Half of the students translated English into Chinese, while the other half translated words in the opposite direction. Only when 60% of the students gave the same translations for a given word were considered as translation equivalents of each other.

Meanwhile, another 20 Chinese learners of English from the same population were recruited to rate the concreteness and familiarity of English words on a five-point scale (1 for “quite abstract” and “very unfamiliar,” and 5 for “quite concrete” and “very familiar”). Finally, these 80 Chinese–English pairs, which were categorized into four different sets: 20 cognate abstract word pairs, 20 cognate concrete word pairs, 20 non-cognate abstract word pairs, and 20 non-cognate concrete word pairs, were chosen for the present experiment. A paired-sample *t*-test was conducted to examine the variables of familiarity and concreteness. An independent-sample *t*-test was conducted to examine the length of English and the stroke of Chinese. In cognate and non-cognate trials, the 80 English targets were matched in subjective familiarity and concreteness [*p*s ≥ 0.347, *Cohen’s d*(s) ≤ 0.142]. The length of the English targets and the number of strokes of the Chinese primes were matched between cognates and non-cognates [*p*s ≥ 0.681, *Cohen’s d*(s) ≤ 0.019]. The concrete words and abstract words were matched in subjective familiarity (*p* = 0.109, *Cohen’s d* = 0.377), length of the English targets and the number of strokes of the Chinese primes [*p*s ≥ 0.571, *Cohen’s d*(s) ≤ 0.058].

As the present study attempts to find out whether phonological similarity and concreteness can affect the magnitudes of priming effects, another 80 words were selected as control primes to constitute the unrelated trials. The control primes were matched with the translation (related) primes in terms of the numbers of characters and strokes (*p* = 0.680, *Cohen’s d* = 0.065) as well as concreteness (*p* = 0.937, *Cohen’s d* = 0.018). In the experiment, the targets were presented under two conditions, the related condition in which the primes and the targets were translations of each other, and the unrelated condition in which the primes and the targets were not related in meaning. Additionally, to complete the yes or no response in the lexical decision, task additional 80 Chinese primes were paired with English pseudowords as targets. The English pseudowords were selected from Macquarie Online Test Interface^1^ or generated by Wuggy (Keuleers and Brysbaert, 2010), and they were pronounceable sequences that followed the rules of English orthography. Their average length was matched with that of real English targets. Examples of the stimuli in the experiment were presented in Table 1.

**TABLE 1:** Stimuli examples in Experiments 1 and 2.

Priming direction	Condition	Prime	Control	Target
L1–L2	Abstract cognates	逻辑	情感	Logic
	Concrete cognates	沙发	肌肉	Sofa
	Abstract non-cognates	心情	本能	Mood
	Concrete non-cognates	裤子	泥沙	Pants
L2–L1				
	Abstract cognates	Logic	Genre	逻辑
	Concrete cognates	Sofa	Coin	沙发
	Abstract non-cognates	Mood	Fate	心情
	Concrete non-cognates	Pants	Scarf	裤子

#### Procedure

**FIGURE 1:**
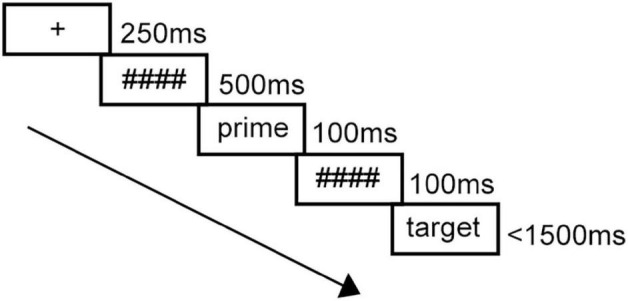
Trial structure of the experiment.

All the participants were tested in front of a computer in a sound attenuated room while the experimenter could monitor the process in another room. The experimental program was designed by E-Prime 3.0. In the experiment, participants needed to respond to 240 trials in total (160 with a word target, 80 with a pseudoword target). Each item was presented at the center of the monitor. First, the fixation point “+” was displayed for 250 ms followed by a row of hash masks (#) for 500 ms. Next, a prime word appeared for 100 ms before it was replaced by the backward mask which lasted 100 ms. The length of pre- and post-masks for Chinese primes was matched with two hash masks for one Chinese character. Then the English target word was presented until the participant made a response, but for no more than 1,500 ms (see Figure 1). Participants were asked to decide whether the target words were real words or not. They could indicate their answers by pressing two keys on the keyboard, “J” or “F.” The assignment of which key represented real words was counterbalanced across participants. There was a random interval of 300–500 ms after each trial. Before the experiment, 12 practice trials were constructed to help participants get familiar with the experimental process. Every target appeared twice, once in the related condition and once in the unrelated condition. The order of the two conditions was counterbalanced for a given target word and the presentation of trials was randomized.

#### Electroencephalogram Recording Procedure

The electroencephalogram (EEG) was recorded from 32 scalp sites by an electrodes cap following a revised standard International 10–20 system. Two electrodes were located next to the canthus to monitor horizontal activity, and vertical eye movement was monitored by another two electrodes next to the left eye up and down. All the data were re-referenced to the mean electric activity of the mastoids. The digitizing computer continuously sampled the EEG at a rate of 1,000 Hz. Scalp electrodes impedances were maintained below 5 kΩ and Bandpass was filtered between 0.01 and 100 Hz. The EEG was collected online and analyzed offline by Neuroscan Curry 8.

### Data Analyses and Results

The entire data of three participants were excluded from analyses in forward translation due to higher error rates (over 30%) in behavioral and ERP analyses. The mean response times and error rates (E%) in Experiment 1 across each experimental condition were presented in Table 2.

**TABLE 2:** Mean RTs/error rates (E%) as a function of translation direction, cognate status and concreteness.

	Translation	Control	Priming effect
**Experiment 1 forward translation (Chinese–English)**			
Abstract cognates	614.1/4.1	701.1/18.0	87.1*/13.9
Concrete cognates	570.3/2.0	650.0/7.0	79.8*/5.0
Abstract non-cognates	604.3/2.0	657.5/6.1	53.3*/4.1
Concrete non-cognates	590.3/1.8	657.0/7.3	66.7*/5.5
**Experiment 2 backward translation (English–Chinese)**			
Abstract cognates	580.0/1.4	602.3/2.3	22.2*/0.9
Concrete cognates	557.6/1.1	589.4/2.3	31.8*/1.1
Abstract non-cognates	564.5/2.0	582.5/2.5	18.1*/0.5
Concrete non-cognates	552.9/0.2	577.9/1.6	25.1*/1.4

*p < 0.05. Significant difference between the translation (related) condition and the control (unrelated) condition.

#### Behavioral Analyses

The mean reaction times (RTs) and error rates (E%) were submitted to a 4 (word type: abstract cognates, concrete cognates, abstract non-cognates and concrete non-cognates) × 2 (relatedness: related and unrelated) design. Repeated-measures analysis of variance (ANOVA) by subjects and univariate ANOVA by items examined translation priming effects of each word type. The Greenhouse–Geisser correction was applied to all repeated-measures with more than one degree of freedom in the numerator in the present study.

The behavioral data on RT analysis showed that there was a significant main effect of word type [*F*_1_(3,63) = 18.304, *p* < 0.001, $\eta_{\text{p}}^{2}$ = 0.466, *F*_2_(3,76) = 4.750, *p* = 0.004, $\eta_{\text{p}}^{2}$ = 0.158], and of relatedness [*F*_1_(1,21) = 85.218, *p* < 0.001, $\eta_{\text{p}}^{2}$ = 0.802, *F*_2_(1,76) = 296.164, *p* < 0.001, $\eta_{\text{p}}^{2}$ = 0.796]. The interaction between word type and relatedness was (marginally) significant [*F*_1_(3,63) = 2.555, *p* = 0.067, $\eta_{\text{p}}^{2}$ = 0.108, *F*_2_(3,76) = 3.192, *p* = 0.028, $\eta_{\text{p}}^{2}$ = 0.112]. Simple effect comparisons showed that the relatedness effects were significant for all word types [*F*_1_s(1,21) ≥ 21.141, *p*s ≤ 0.001, $\eta_{\text{p}}^{2}$s ≥ 0.502, *F*_2_s(1,76) ≥ 40.905, *p*s ≤ 0.001, $\eta_{\text{p}}^{2}$s ≥ 0.350]. The response times of the unrelated condition were much longer than that of the related condition.

For the analysis of error rate, there was a significant main effect of word type [*F*_1_(3,63) = 18.563, *p* < 0.001, $\eta_{\text{p}}^{2}$ = 0.469, *F*_2_(3,76) = 8.086, *p* < 0.001, $\eta_{\text{p}}^{2}$ = 0.242], and of relatedness [*F*_1_(1,21) = 20.171, *p* < 0.001, $\eta_{\text{p}}^{2}$ = 0.490, *F*_2_(1,76) = 29.949, *p* < 0.001, $\eta_{\text{p}}^{2}$ = 0.283]. The interaction between word type and relatedness was also significant [*F*_1_(3,63) = 12.380, *p* < 0.001, $\eta_{\text{p}}^{2}$ = 0.371, *F*_2_(3,76) = 11.102, *p* < 0.001, $\eta_{\text{p}}^{2}$ = 0.305]. Simple effect comparisons revealed (marginally) significant differences for relatedness factor in all word types in analyses by subjects [*F*_1_s(1,21) ≥ 4.074, *p*s ≤ 0.057, $\eta_{\text{p}}^{2}$s ≥ 0.162] and by items [*F*_2_s(1,76) ≥ 4.364, *p*s ≤ 0.040, $\eta_{\text{p}}^{2}$s ≥ 0.054], except for the cognate concrete words which failed to reach significance by items [*F*_2_(1,76) = 1.017, *p* = 0.316, $\eta_{\text{p}}^{2}$ = 0.013)].

Following the previous study (Ferré et al., 2017), the magnitudes of priming effects in the present study were calculated by subtracting the response times and error rates of the related conditions from the unrelated conditions for detecting the greater phonological and conceptual overlaps in translation pairs than unrelated pairs in DFM (de Groot, 1992; van Hell and de Groot, 1998). Separate ANOVAs on the magnitude of priming effects were conducted for RT and E% data with the independent factors of cognate status (cognate and non-cognate) and concreteness (abstract and concrete) by subjects (*F*_1_) and by items (*F*_2_).

The behavioral data on RT analysis showed that there was a significant effect for cognate status [*F*_1_(1,21) = 6.480, *p* = 0.019, $\eta_{\text{p}}^{2}$ = 0.236, *F*_2_(1,76) = 7.900, *p* = 0.006, $\eta_{\text{p}}^{2}$ = 0.094], reflecting that the priming magnitude of cognates was greater than that of non-cognates. The main effect of concreteness was not significant [*F*_1_(1,21) = 0.172, *p* = 0.682, $\eta_{\text{p}}^{2}$ = 0.008, *F*_2_(1,76) = 0.134, *p* = 0.716, $\eta_{\text{p}}^{2}$ = 0.002]. The interaction between cognate status and concreteness failed to reach the statistical significance [*F*_1_(1,21) = 0.898, *p* = 0.354, $\eta_{\text{p}}^{2}$ = 0.041, *F*_2_(1,76) = 1.541, *p* = 0.218, $\eta_{\text{p}}^{2}$ = 0.020].

E% data were submitted to the same analysis. There was a significant main effect for cognate status [*F*_1_(1,21) = 13.174, *p* = 0.002, $\eta_{\text{p}}^{2}$ = 0.385, *F*_2_(1,76) = 4.785, *p* = 0.032, $\eta_{\text{p}}^{2}$ = 0.059], indicating that the error rate of cognates was higher than non-cognates. The main effect of concreteness was (marginally) significant [*F*_1_(1,21) = 16.047, *p* = 0.001, $\eta_{\text{p}}^{2}$ = 0.433, *F*_2_(1,76) = 3.100, *p* = 0.082, $\eta_{\text{p}}^{2}$ = 0.039], and the error rate of abstract words was significantly higher than that of concrete words. The interaction between cognate status and concreteness was significant [*F*_1_(1,21) = 9.682, *p* = 0.005, $\eta_{\text{p}}^{2}$ = 0.316, *F*_2_(1,76) = 5.764, *p* = 0.019, $\eta_{\text{p}}^{2}$ = 0.070]. Simple effect comparisons revealed that the error rate in abstract cognates was significantly higher than that in abstract non-cognates [*F*_1_(1,21) = 16.733, *p* = 0.001, $\eta_{\text{p}}^{2}$ = 0.443, *F*_2_(1,76) = 10.526, *p* = 0.002, $\eta_{\text{p}}^{2}$ = 0.122]. In addition, the error rate in abstract cognates was significantly higher than that in concrete cognates [*F*_1_(1,21) = 22.234, *p* < 0.001, $\eta_{\text{p}}^{2}$ = 0.514, *F*_2_(1,76) = 8.659, *p* = 0.004, $\eta_{\text{p}}^{2}$ = 0.102].

#### Electroencephalogram Data Analyses

**FIGURE 2:**
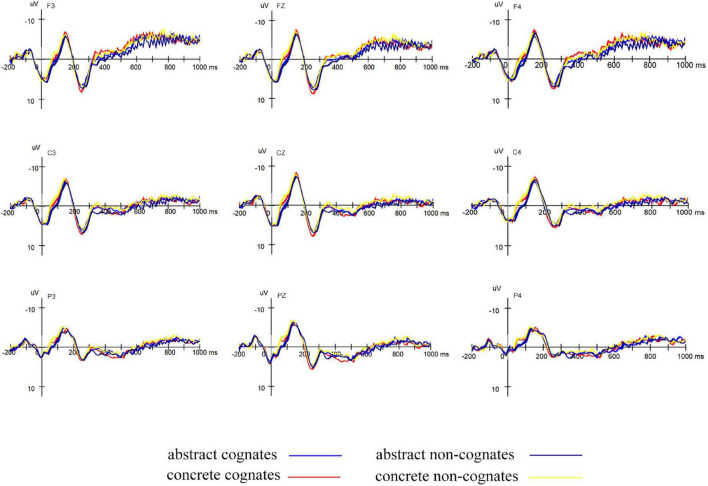
Grand average ERPs elicited by targets primed by abstract cognates, abstract non-cognates, concrete cognates, and concrete non-cognates in L1–L2 translation direction.

As shown in Figure 2, visual inspection of the grand mean ERP components elicited by target presentation revealed a negative peak in the 100–200 ms post-stimulus time window (N150), a positive peak in the 200–350 ms time window (P250), and a negative peak in the 350–550 ms time window (N400). In the previous studies, a component in the time window of 100–200 ms was usually considered as the mapping of visual features onto prelexical features in the word-base process (Holcomb and Grainger, 2006; Hoshino et al., 2010). In the present study, N150 can be regarded to reflect the processing of phonological information, and the mean amplitudes of the electrodes F3, Fz, F4, C3, Cz, C4, P3, Pz, P4, O1, Oz, and O2 in the 100–200 ms time window for each participant in all conditions were extracted based on Kong et al. (2010). According to Kutas and Federmeier (2011), the N400 component was an indicator of semantic processing, largest over centro-parietal sites. Therefore, we extracted the mean amplitudes of the electrodes C3, Cz, C4, CP3, CPz, CP4, P3, Pz, and P4 in the 350–550 ms time window. In a Chinese–English non-cognates translation priming experiment, Chen et al. (2020) identified N300 component between P200 and P400 as the index of the morphological-semantic interface. Therefore, as an ERP component between N150 and N400, the present P250 component was thought to be an index of the phonological-semantic interface, and the mean amplitudes of the electrodes F3, Fz, F4, C3, Cz, and C4 in the 200–350 ms time window were extracted.

We first examined whether there were translation priming effects of each word type in forward translation.

N150: The mean amplitudes were subjected to a 4 (word type: abstract cognates, concrete cognates, abstract non-cognates and concrete non-cognates) × 2 (relatedness: related and unrelated) × 12 (electrode: F3, Fz, F4, C3, Cz, C4, P3, Pz, P4, O1, Oz, and O2) repeated-measure ANOVA. Statistical analysis showed that there was no main effect of either word type [*F*(3,63) = 0.798, *p* = 0.471, $\eta_{\text{p}}^{2}$ = 0.037], or relatedness [*F*(1,21) = 0.001, *p* = 0.972, $\eta_{\text{p}}^{2}$ < 0.001]. Additionally, the interaction between word type and relatedness was not significant [*F*(3,63) = 0.494, *p* = 0.688, $\eta_{\text{p}}^{2}$ = 0.023]. Planned comparisons indicated that none of the word types showed significant relatedness effects (*p*s ≥ 0.484, $\eta_{\text{p}}^{2}$s ≤ 0.024).

P250: The analysis in the 200–350 ms time window with six electrodes (F3, Fz, F4, C3, Cz, and C4) showed that there was no main effect of either word type [*F*(3,63) = 0.800, *p* = 0.470, $\eta_{\text{p}}^{2}$ = 0.037], or relatedness [*F*(1,21) = 0.008, *p* = 0.931, $\eta_{\text{p}}^{2}$ < 0.001]. In addition, the interaction between word type and relatedness was not significant [*F*(3,63) = 0.639, *p* = 0.593, $\eta_{\text{p}}^{2}$ = 0.030]. Planned comparisons indicated that none of the word types showed significant relatedness effects. (*p*s ≥ 0.304, $\eta_{\text{p}}^{2}$s ≤ 0.050).

N400: The analysis in the 350–550 ms time window with 9 electrodes (C3, Cz, C4, CP3, CPz, CP4, P3, Pz, and P4) showed that there was a significant main effect of word type [*F*(3,63) = 3.310, *p* = 0.035, $\eta_{\text{p}}^{2}$ = 0.136], and of relatedness [*F*(1,21) = 20.770, *p* < 0.001, $\eta_{\text{p}}^{2}$ = 0.497]. The interaction between word type and relatedness was not significant [*F*(3,63) = 0.396, *p* = 0.681, $\eta_{\text{p}}^{2}$ = 0.018]. Planned comparisons showed that the relatedness effects were significant for all word types [*F*s(1,21) ≥ 5.262, *p*s ≤ 0.032, $\eta_{\text{p}}^{2}$s ≥ 0.200], and related condition elicited significantly larger N400 than unrelated condition.

Then, we examined whether cross-script phonological similarity and concreteness could elicit greater priming effects in forward translation. The difference waves (the mean amplitudes of the unrelated condition minus the mean amplitudes of the related condition) of the same electrodes as the three ERP components mentioned above in three time windows (100–200, 200–350, and 350–550 ms) were submitted to statistical analyses, respectively.

100–200 ms: The difference waves were subjected to a 2 (cognate status: cognate and non-cognate) × 2 (concreteness: abstract and concrete) × 12 (electrode: F3, Fz, F4, C3, Cz, C4, P3, Pz, P4, O1, Oz, and O2) repeated-measure ANOVA. Statistical analysis showed that there was no main effect of either cognate status [*F*(1,21) = 0.016, *p* = 0.901, $\eta_{\text{p}}^{2}$ = 0.001], or concreteness [*F*(1,21) = 0.890, *p* = 0.356, $\eta_{\text{p}}^{2}$ = 0.041]. The interaction between cognate status and concreteness was not significant either [*F*(1,21) = 1.866, *p* = 0.186, $\eta_{\text{p}}^{2}$ = 0.082].

200–350 ms: The analysis in the 200–350 ms time window with six electrodes (F3, Fz, F4, C3, Cz, and C4) showed that there was no main effect of either cognate status [*F*(1,21) = 1.012, *p* = 0.326, $\eta_{\text{p}}^{2}$ = 0.046], or concreteness [*F*(1,21) = 2.158, *p* = 0.157, $\eta_{\text{p}}^{2}$ = 0.093]. Additionally, the interaction between cognate status and concreteness was not significant [*F*(1,21) = 0.024, *p* = 0.878, $\eta_{\text{p}}^{2}$ = 0.001].

350–550 ms: The analysis in the 350–550 ms time window with nine electrodes (C3, Cz, C4, CP3, CPz, CP4, P3, Pz, and P4) showed that there was no main effect of either cognate status [*F*(1,21) = 1.268, *p* = 0.273, $\eta_{\text{p}}^{2}$ = 0.057], or concreteness [*F*(1,21) = 0.062, *p* = 0.806, $\eta_{\text{p}}^{2}$ = 0.003]. In addition, the interaction between cognate status and concreteness was not significant [*F*(1,21) = 0.004, *p* = 0.952, $\eta_{\text{p}}^{2}$ < 0.001].

In summary, the results of Experiment 1 indicated that the translation priming effects from Chinese to English were reflected in RT data, E% data and N400 component. The priming effects of cognate status were shown in RT data and E% data, whereas the priming effects of concreteness and interaction between cognate status and concreteness were only sensitive to E% data. No ERP evidence was found for the greater priming effects of cognate status and concreteness in forward translation since no main effects nor interaction effects in the three time windows (100–200, 200–350, and 350–550 ms) were observed on the difference waves, respectively.

## Experiment 2: L2–L1 (English–Chinese Backward Translation)

### Methods

Experiment 2 explored the role of phonology as well as concreteness effects for Chinese learners of English with a lexical decision task in the masked translation priming paradigm in the L2–L1 translation direction.

#### Participants

This experiment had the same participants as Experiment 1.

#### Materials

The experimental materials were the same as in Experiment 1 except for the priming direction in which the primes were English and the targets were presented in Chinese. In the backward direction, translation primes and control primes (unrelated primes) were matched in length, concreteness, and familiarity [*p*s ≥ 0.119, *Cohen’s d*(s) ≤ 0.161]. In addition, there were also 80 Chinese pseudowords, which were meaningless words comprised of two or three characters. The real words and pseudowords were also matched in the number of strokes. Examples of the stimuli in the experiment were presented in Table 1.

#### Procedure

The procedure of Experiment 2 replicates the experimental procedure of Experiment 1, except that the length of pre- and post-masks for English primes was matched with one hash mask for one English letter. Then the Chinese target word was presented until the participant made a response, but for no more than 1,500 ms (see Figure 1). There was 1-h interval between Experiments 2 and 1, during which an experiment unrelated to the present two experiments was conducted in order to avoid the mutual influence of the present two experiments.

#### Electroencephalogram Recording Procedure

The EEG recording procedure was the same as in Experiment 1.

### Data Analyses and Results

The three participants whose data were deleted in Experiment 1 due to the high error rates (over 30%), and their data in Experiment 2 were also discarded due to high error rates (over 30%) in data analyses. The mean RTs and error rates in Experiment 2 across each experimental condition are presented in Table 2.

#### Behavioral Analyses

Similar to Experiment 1, the mean reaction times and error rates were submitted to 4 (word type: abstract cognates, concrete cognates, abstract non-cognates, and concrete non-cognates) × 2 (relatedness: related and unrelated) separate ANOVAs by subjects and by items to examine backward translation priming effects of each word type.

The data analysis of RT showed that there was a significant main effect of word type [*F*_1_(3,63) = 14.080, *p* < 0.001, $\eta_{\text{p}}^{2}$ = 0.401, *F*_2_(3,76) = 5.709, *p* = 0.001, $\eta_{\text{p}}^{2}$ = 0.184], and of relatedness [*F*_1_ (1,21) = 44.362, *p* < 0.001, $\eta_{\text{p}}^{2}$ = 0.679, *F*_2_(1,76) = 73.623, *p* < 0.001, $\eta_{\text{p}}^{2}$ = 0.492]. The interaction between word type and relatedness failed to reach significance [*F*_1_(3,63) = 0.903, *p* = 0.434, $\eta_{\text{p}}^{2}$ = 0.041, *F*_2_(3,76) = 1.046, *p* = 0.377, $\eta_{\text{p}}^{2}$ = 0.040]. Planned comparisons revealed that there were significant relatedness effects for all word types [*F*_1_s(1,21) ≥ 7.089, *p*s ≤ 0.015, $\eta_{\text{p}}^{2}$s ≥ 0.252, *F*_2_s(1,76) ≥ 10.160, *p*s ≤ 0.020, $\eta_{\text{p}}^{2}$s ≥ 0.118], with longer response times in unrelated condition than that in related condition.

For the error rate data, the main effect of relatedness was significant by items [*F*_2_(1,76) = 5.609, *p* = 0.020, $\eta_{\text{p}}^{2}$ = 0.069], but not significant by subjects [*F*_1_(1,21) = 0.606, *p* = 0.445, $\eta_{\text{p}}^{2}$ = 0.028]. There was no significant main effect of word type [*F*_1_(3,63) = 1.035, *p* = 0.367, $\eta_{\text{p}}^{2}$ = 0.047, *F*_2_(3,76) = 1.903, *p* = 0.136, $\eta_{\text{p}}^{2}$ = 0.070]. The interaction between word type and relatedness failed to reach significance [*F*_1_(3,63) = 0.287, *p* = 0.712, $\eta_{\text{p}}^{2}$ = 0.013, *F*_2_(3,76) = 0.226, *p* = 0.878, $\eta_{\text{p}}^{2}$ = 0.009].

The magnitudes of priming effects were submitted to 2 (cognate status: cognates and non-cognates) × 2 (concreteness: abstract words and concrete words) separate ANOVAs by subjects and by items to examine the cognate effects and concreteness effects.

The data analyses of RT showed that no significant main effects nor interaction effects were found by subjects (*p*s ≥ 0.232, $\eta_{\text{p}}^{2}$s ≤ 0.067) and by items (*p*s ≥ 0.219, $\eta_{\text{p}}^{2}$s ≤ 0.020).

Meanwhile, the same analysis was conducted on the data of error rate, and no significant main effects nor interaction effects were found in analyses by subjects (*p*s ≥ 0.303, $\eta_{\text{p}}^{2}$s ≤ 0.050) and by items (*p*s ≥ 0.488, $\eta_{\text{p}}^{2}$s ≤ 0.006).

#### Electroencephalogram Data Analyses

**FIGURE 3:**
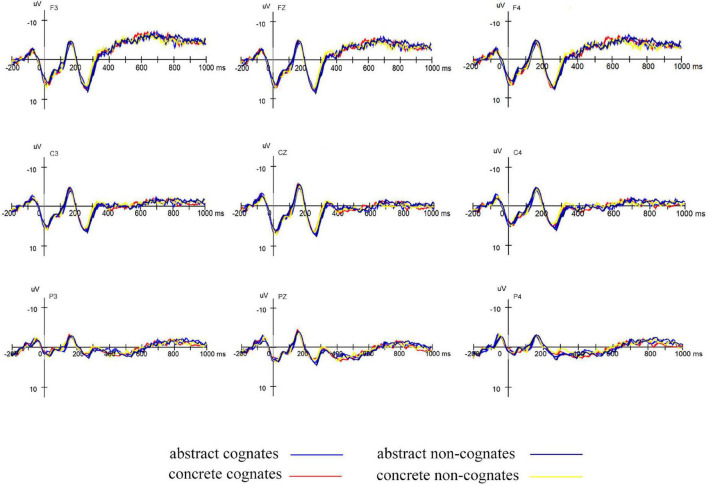
Grand average ERPs elicited by targets primed by abstract cognates, abstract non-cognates, concrete cognates, and concrete non-cognates in L2–L1 translation direction.

As shown in Figure 3, visual inspection of the grand mean ERP components elicited by target presentation revealed a negative peak in the 100–200 ms time window (N150), a positive peak in the 200–350 ms time window (P250), and a negative peak in the 350–550 ms time window (N400). We selected the same electrodes and conducted the same statistical analyses as in Experiment 1.

We first examined whether there were translation priming effects of each word type in backward translation.

N150: The mean amplitudes were subjected to a 4 (word type: abstract cognates, concrete cognates, abstract non-cognates and concrete non-cognates) × 2 (relatedness: related and unrelated) × 12 (electrode: F3, Fz, F4, C3, Cz, C4, P3, Pz, P4, O1, Oz, and O2) repeated-measure ANOVA. Statistical analysis showed that there was no main effect of either word type [*F*(3,63) = 0.787, *p* = 0.496, $\eta_{\text{p}}^{2}$ = 0.036], or relatedness [*F*(1,21) = 2.122, *p* = 0.160, $\eta_{\text{p}}^{2}$ = 0.092]. There was a significant interaction between word type and relatedness [*F*(3,63) = 4.041, *p* = 0.015, $\eta_{\text{p}}^{2}$ = 0.161]. Simple effect comparisons revealed that related abstract cognates elicited significantly larger N150 than unrelated abstract cognates [*F*(1,21) = 9.122, *p* = 0.007, $\eta_{\text{p}}^{2}$ = 0.303], and the mean amplitudes were −1.862 and −1.016 (μV, respectively; that related concrete non-cognates elicited marginally larger N150 than unrelated concrete non-cognates [F (1,21) = 4.015, *p* = 0.058, $\eta_{p}^{2}=0.161$, and the mean amplitudes were −1.682 and −1.033 μV, respectively. There were no significant effects of relatedness for the concrete cognate and abstract non-cognates (*p*s ≥ 0.107, $\eta_{p}^{2}$s ≤ 0.119).

P250: The analysis in the 200–350 ms time window with six electrodes (F3, Fz, F4, C3, Cz, and C4) showed that there was a main effect of word type [*F*(3,63) = 4.551, *p* = 0.009, $\eta_{\text{p}}^{2}$ = 0.178]. There was no significant main effect of relatedness [*F*(1,21) = 1.760, *p* = 0.199, $\eta_{\text{p}}^{2}$ = 0.077]. The interaction between word type and relatedness was not significant [*F*(3,63) = 0.550, *p* = 0.611, $\eta_{\text{p}}^{2}$ = 0.026]. Planned comparisons indicated that none of the word types showed significant relatedness effects (*p*s ≥ 0.125, $\eta_{\text{p}}^{2}$s ≤ 0.136).

N400: The analysis in the 350–550 ms time window with nine electrodes (C3, Cz, C4, CP3, CPz, CP4, P3, Pz, and P4) revealed no significant main effect of either word type [*F*(3,63) = 0.928, *p* = 0.418, $\eta_{\text{p}}^{2}$ = 0.042], or relatedness [*F*(1,21) = 1.685, *p* = 0.208, $\eta_{\text{p}}^{2}$ = 0.074]. Additionally, the interaction between word type and relatedness was not significant [*F*(3,63) = 1.271, *p* = 0.293, $\eta_{\text{p}}^{2}$ = 0.057]. The planned comparisons revealed that related concrete cognates elicited marginally significantly larger N400 than unrelated concrete cognates [*F*(1,21) = 3.344, *p* = 0.082, $\eta_{\text{p}}^{2}$ = 0.137], and the mean amplitudes were 1.343 and 0.635 (μV, respectively. There were no significant effects of relatedness for the abstract cognates, concrete non-cognates and abstract non-cognates [*p*s ≥ 0.172, $\eta_{\text{p}}^{2}$s ≤ 0.087].

Then, we examined whether cross-script phonological similarity and concreteness could elicit greater priming effects in backward translation. The difference waves (the mean amplitude of the unrelated condition minus the mean amplitude of the related condition) of the same electrodes as the three ERP components mentioned above in three time windows (100–200, 200–350, and 350–550 ms) were submitted to statistical analyses, respectively.

100–200 ms: The difference waveforms were subjected to a 2 (cognate status: cognate, non-cognates) × 2 (concreteness: abstract and concrete) × 12 (electrode: F3, Fz, F4, C3, Cz, C4, P3, Pz, P4, O1, Oz, and O2) repeated-measure ANOVA. Statistical analysis showed that there was no significant main effect of either cognate status [*F*(1,21) = 2.134, *p* = 0.159, $\eta_{\text{p}}^{2}$ = 0.092], or concreteness [*F*(1,21) = 0.355, *p* = 0.558, $\eta_{\text{p}}^{2}$ = 0.017]. There was a significant interaction between cognate status and concreteness [*F*(1,21) = 14.061, *p* = 0.001, $\eta_{\text{p}}^{2}$ = 0.401]. Simple effect comparisons revealed that abstract non-cognates produced significantly smaller difference waves than concrete non-cognates [*F*(1,21) = 7.270, *p* = 0.014, $\eta_{\text{p}}^{2}$ = 0.257], and the mean amplitudes were −0.573 and 0.649 μV, respectively; abstract cognates produced significantly larger difference waves than abstract non-cognates [*F*(1,21) = 16.881, *p* = 0.001, $\eta_{\text{p}}^{2}$ = 0.446], and the mean amplitudes were 0.846 and −0.573 μV, respectively.

200–350 ms: The analysis in the 200–350 ms time window with six electrodes (F3, Fz, F4, C3, Cz, and C4) showed that there was no main effect of either cognate status [*F*(1,21) = 0.168, *p* = 0.686, $\eta_{\text{p}}^{2}$ = 0.008], or concreteness [*F*(1,21) = 0.653, *p* = 0.428, $\eta_{\text{p}}^{2}$ = 0.030]. In addition, the interaction between cognate status and concreteness was not significant [*F*(1,21) = 0.895, *p* = 0.355, $\eta_{\text{p}}^{2}$ = 0.041].

350–550 ms: The analysis in the 350–550 ms time window with nine electrodes (C3, Cz, C4, CP3, CPz, CP4, P3, Pz, and P4) showed that there was no main effect of either cognate status [*F*(1,21) = 2.812, *p* = 0.108, $\eta_{\text{p}}^{2}$ = 0.118], or concreteness [*F*(1,21) = 0.458, *p* = 0.506, $\eta_{\text{p}}^{2}$ = 0.021]. Additionally, the interaction between cognate status and concreteness was not significant [*F*(1,21) = 0.151, *p* = 0.702, $\eta_{\text{p}}^{2}$ = 0.007].

In summary, the results of Experiment 2 demonstrated that backward translation priming effects were obtained in RT data for each type of words. The ERP evidence for translation priming effects was obtained in terms of the N150 for abstract cognates and concrete non-cognates, as well as the N400 for concrete cognates. The interaction effect between cognate status and concreteness in the time window of 100–200 ms indicated that concrete non-cognates had greater priming effects than abstract non-cognates and abstract cognates had greater priming effects than abstract non-cognates.

## Joint Analyses

In order to investigate whether there existed the asymmetry of translation direction in terms of the priming effect magnitudes between forward translation and backward translation, joint analyses were conducted by comparing behavioral and ERP data in Experiment 1 and Experiment 2.

### Behavioral Analyses

Separate ANOVAs were performed for the magnitudes of priming with the factors of translation direction (2: forward direction and backward direction) and word type (4: abstract cognates, concrete cognates, abstract non-cognates, and concrete non-cognates) for the RT to examine the existence of translation priming asymmetry. The main effect of direction reached significance [*F*_1_(1,21) = 24.387, *p* < 0.001, $\eta_{\text{p}}^{2}$ = 0.537, *F*_2_(1,152) = 88.496, *p* < 0.001, $\eta_{\text{p}}^{2}$ = 0.368], and there were larger priming effects in forward translation than in backward translation. The main effect of word type was also significant [*F*_1_(3,63) = 3.155, *p* = 0.032, $\eta_{\text{p}}^{2}$ = 0.131, *F*_2_(3,152) = 3.435, *p* = 0.019, $\eta_{\text{p}}^{2}$ = 0.063]. The interaction between translation direction and word type failed to reach significance [*F*_1_(3,63) = 0.850, *p* = 0.458, $\eta_{\text{p}}^{2}$ = 0.039, *F*_2_(3,152) = 1.592, *p* = 0.194, $\eta_{\text{p}}^{2}$ = 0.030]. Planned comparisons revealed that the direction effects were significant for all word types [*F*_1_s(1,21) ≥ 5.600, *p*s ≤ 0.028, $\eta_{\text{p}}^{2}$s ≥ 0.211, *F*_2_s(1,76) ≥ 12.228, *p*s ≤ 0.001, $\eta_{\text{p}}^{2}$s ≥ 0.074], and that larger priming effects were found for each word type in forward translation than in backward translation.

For the analysis of error data, the main effect of direction was significant [*F*_1_(1,21) = 16.179, *p* = 0.001, $\eta_{\text{p}}^{2}$ = 0.435, *F*_2_(1,152) = 28.954, *p* < 0.001, $\eta_{\text{p}}^{2}$ = 0.160], and the error rate was higher in forward translation than that in backward translation. The main effect of word type was also significant [*F*_1_(3,63) = 11.709, *p* < 0.001, $\eta_{\text{p}}^{2}$ = 0.358, *F*_2_(3,152) = 4.015, *p* = 0.009, $\eta_{\text{p}}^{2}$ = 0.073]. There was a significant interaction between translation direction and word type [*F*_1_(3,63) = 6.251, *p* = 0.004, $\eta_{\text{p}}^{2}$ = 0.229, *F*_2_(3,152) = 3.978, *p* = 0.009, $\eta_{\text{p}}^{2}$ = 0.073]. Simple effect comparisons revealed that priming effects of all word types were (marginally) significantly larger in forward translation than those in backward translation in analyses by participants [*F*_1_s(1,21) ≥ 4.841, *p*s ≤ 0.039, $\eta_{\text{p}}^{2}$s ≥ 0.187] except for concrete non-cognates [*F*_1_(1,21) = 2.872, *p* = 0.105, $\eta_{\text{p}}^{2}$ = 0.120], and by items [*F*_2_s(1,152) ≥ 2.870, *p*s ≤ 0.092, $\eta_{\text{p}}^{2}$s ≥ 0.019] except for abstract non-cognates [*F*_2_(1,152) = 2.542, *p* = 0.113, $\eta_{\text{p}}^{2}$ = 0.016].

### Electroencephalogram Data Analyses

To examine the existence of the priming asymmetry between forward translation and backward translation, we compared the ERP difference waves in the time windows of 100–200 ms, 200–350 ms, and 350–550 ms between the two directions.

100–200 ms: The difference waves were subjected to a 2 (translation direction: forward and backward) × 4 (word type: abstract cognates, concrete cognates, abstract non-cognates, and concrete non-cognates) × 12 (electrode: F3, Fz, F4, C3, Cz, C4, P3, Pz, P4, O1, Oz, and O2) repeated-measure ANOVA. Statistical analysis showed that there was a significant main effect of word type [*F*(3,63) = 4.136, *p* = 0.011, $\eta_{\text{p}}^{2}$ = 0.165]. The main effect of direction was not significant [*F*(1,21) = 0.987, *p* = 0.332, $\eta_{\text{p}}^{2}$ = 0.045]. The interaction between translation direction and word type was not significant [*F*(3,63) = 0.733, *p* = 0.510, $\eta_{\text{p}}^{2}$ = 0.034]. Planned comparisons revealed that abstract cognates in forward translation elicited marginally significantly smaller difference waves than abstract cognates in backward translation [*F*(1,21) = 3.338, *p* = 0.082, $\eta_{\text{p}}^{2}$ = 0.137], and the mean amplitudes were 0.072 and 0.846 μV, respectively. There were no translation direction effects in terms of the concrete cognates, abstract non-cognates and concrete non-cognates (*p*s ≥ 0.662, $\eta_{\text{p}}^{2}$s ≤ 0.009).

200–350 ms: The analysis in the 200–350 ms time window with six electrodes (F3, Fz, F4, C3, Cz, and C4) presented no significant main effect of either direction [*F*(1,21) = 0.854, *p* = 0.366, $\eta_{\text{p}}^{2}$ = 0.039], or word type [*F*(3,63) = 0.993, *p* = 0.401, $\eta_{\text{p}}^{2}$ = 0.045]. Additionally, the interaction between translation direction and word type was not significant [*F*(3,63) = 0.580, *p* = 0.621, $\eta_{\text{p}}^{2}$ = 0.027]. Planned comparisons indicated that none of the word types showed significant direction effects (*p*s ≥ 0.182, $\eta_{\text{p}}^{2}$s ≤ 0.083).

350–550 ms: The analysis in the 350–550 ms time window with nine electrodes (C3, Cz, C4, CP3, CPz, CP4, P3, Pz, and P4) revealed a significant main effect of direction [*F*(1,21) = 20.726, *p* < 0.001, $\eta_{\text{p}}^{2}$ = 0.497]. The main effect of word type was not significant [*F*(3,63) = 1.549, *p* = 0.217, $\eta_{\text{p}}^{2}$ = 0.069]. The interaction between translation direction and word type was not significant [*F*(3,63) = 0.150, *p* = 0.903, $\eta_{\text{p}}^{2}$ = 0.007]. Planned comparisons revealed that the asymmetry of the priming effects between forward translation and backward translation existed in all word types [*F*s(1,21) ≥ 4.564, *p*s ≤ 0.045, $\eta_{\text{p}}^{2}$s ≥ 0.179] with larger priming effects in forward translation than in backward translation.

In summary, the differences between L1–L2 direction and L2–L1 direction in behavioral data analyses reflected greater priming effects in forward translation than in backward translation. Meanwhile, the translation priming asymmetry was observed in terms of smaller priming effect for forward translation than for backward translation in the time window of 100–200 ms for abstract cognates, and in terms of larger priming effects for forward translation than for backward translation in the time window of 350–550 ms for each type of words.

## General Discussion

The present study investigated the impact of cross-script cognate phonological activation and concreteness with Chinese–English cognates that shared similar pronunciation and concept simultaneously in masked translation priming paradigm based on ERP technology. The roles of cross-script cognate status and concreteness were investigated in forward translation and backward translation throughout the analyses of the behavioral data and ERP data. The results of behavioral data analyses showed the translation priming effects for four types of word in both translation directions, and greater priming effects were observed for cross-script cognate status with larger priming effects for cognates than for non-cognates in forward translation, but not in backward translation, and the translation priming asymmetry was found. However, the ERP evidence from the results of data analyses in Experiment 1, Experiment 2 and joint analyses showed different influences of cognate status and concreteness on cross-script language processing, and confirmed the existence of the asymmetry of translation directions indicated by different ERP indices. As a whole, N400 effect was found to be closely related to cross-script cognate status advantage and the role of concreteness effect in forward translation, and in the reverse direction N150 and N400 effects were related to the roles of cross-script cognate effect and concreteness effect. In the time window of 100–200 ms for backward translation, we found greater priming effects in concrete words than in abstract words for non-cognates and greater priming effects in cognates than in non-cognates for abstract words. Meanwhile, the asymmetry of translation directions was observed with smaller priming effects in forward translation than in backward translation in the time window of 100–200 ms for abstract cognates, and with larger priming effects in forward translation than in backward translation in the time window of 350–550 ms for each type of words.

### Priming Effects of Cross-Script Cognates

In the previous studies of cognate status, phonological information failed to disentangle from orthographic similarity within same-script languages. Chinese–English cognates that shared similar semantic and phonological representation without orthographic links showed strong evidence for the phonological advantage with respect to cognate status for cross-script languages in the present study.

In the present study, priming effects of cognate status were observed in the N150 in backward translation, and greater priming effects of cross-script cognate status in cognates than in non-cognates for abstract words were found in the time window of 100–200 ms also in backward translation. On the contrary, neither N150 for priming effects of Chinese–English translation nor greater priming effects of phonological information between cognates and non-cognates in the time window of 100–200 ms were found in forward translation. And the translation asymmetry caused by the priming effects of cross-script cognates was indicated by larger amplitudes in the time window 100–200 ms for backward translation than for forward translation. The discrepancy in cognate status due to phonological similarity reflected by N150 component might be interpreted as an indicator of phonological processing during sub-lexical phase since this component was regarded as the mapping of visual features onto prelexical features during word-base process in other studies (Holcomb and Grainger, 2006; Hoshino et al., 2010). The results in backward translation provided the evidence for more phonological overlaps in cognates than in non-cognates.

On the other hand, no ERP evidence was observed for priming effects of Chinese–English translation or for greater priming effects of phonological information between cognates and non-cognates in forward translation. The results might show that the phonological priming effects between cross-script cognates and non-cognates with respect to their corresponding translation equivalents keep similar in forward translation (from L1 Chinese to L2 English). It can be found that the present findings extend the cognate hypothesis stated in DFM (de Groot, 1992; van Hell and de Groot, 1998). While DFM emphasizes the importance of the semantic features in bilingual mental lexicon, it pays less attention to other linguistic features such as phonological features, orthographic features. More phonological features are activated for cross-script cognates than for cross-script non-cognates in masked translation priming paradigm. More activated phonological features in Chinese–English mental lexicon lead to greater phonological priming effects in English–Chinese (L2–L1) priming pairs, not in Chinese–English (L1–L2) priming pairs. The English learning environment for Chinese learners of English may account for the lack of role of phonological similarity in L1–L2 translation. In English classroom, English learners are usually taught to learn L2 English words by remembering their Chinese equivalents, not vice versa. Thus a more frequent repetition from English to Chinese, not from Chinese to English may form a strong phonological activation for English. Therefore, compared with Chinese primes in the L1–L2 direction, English primes as phonograms in the L2–L1 direction gave more direct prompt to activate phonological representation of the target. Meanwhile, the results of the present study are in line with other empirical studies. For example, Zhang et al. (2018) examined the translation priming for cross-script cognates within behavioral data and found that in the L1–L2 priming direction, there was no priming advantage for cognates over non-cognates, and both L1–L2 cognate and non-cognate primes similarly facilitated L2 word recognition and that in the L2–L1 priming direction, only cognate primes facilitated L1 word processing while non-cognates primes failed to generate priming effects. Therefore, the present findings partly support RHM (Kroll and Stewart, 1994) in terms of the weak link from the L1–L2 direction and a strong link from the L2–L1 direction.

The BIA+ model assumes that the appearance of primes leads to activation of phonology, which could render the phonological representations of the targets more easily activated (if the prime and target have phonological similarity). Thus, the pre-activated phonology could accelerate the process of word recognition. There are two routes in the BIA+ model when the lexical phonology is activated, the lexical route and the prelexical route. In the former, activation spreads from sub-lexical orthography to lexical orthography and then to lexical phonology, whereas in the latter, sub-lexical orthography activates sub-lexical phonology which subsequently activates lexical phonology (Dijkstra and van Heuven, 2002). It is possible for phonological activation to occur in the recognition of alphabetical languages since they have regular grapheme-to-phoneme conversion rules. However, in the present cross-script study, the phonological priming effects occurred in the time window of 100–200 ms for abstract words. This time course is ahead of the modulation in 200–250 ms proposed by Ando et al. (2015) in spite of different translation directions. After all, the activation of lexical stage could not accomplish as early as 250 ms after the onset of the stimuli during masked onset priming (Jouravlev et al., 2014). In addition, it has been suggested that N150 component might be interpreted as the sub-lexical phase of lexical processing in mental lexicon in which phonemes or graphemes are activated. Thus the N150 might result from the priming effects that occurred at prelexical stage due to the similar phonological activation in the lexical decision task. Pinyin, a system of Romanized spelling which describes how each Chinese character is pronounced, is in daily use for students in China mainland (for example, typing). Zhou et al. (2010) argued that the pinyin of a given Chinese word could have orthographic overlap with its phonologically similar English word. For instance, “dao” is the Chinese character “道” in pinyin, and there are two overlapping letters in “dao” and its phonologically similar English word “door.” This explanation is also applicable to the present study in that most pinyin of the loan words and their English equivalents are similar to some degree. The processing for the pinyin of “nacui” (“纳粹” in Chinese) was accelerated by its English translation “Nazi” with greater phonological overlap as a prime at the sub-lexical processing phase.

### Priming Effects of Concreteness

The priming effects of concreteness were observed in N400 component in terms of translation priming effects for the four types of words in forward translation, and for cognate concrete words in backward translation, and also in larger priming effects in forward translation than in backward translation in the time window of 350–550 ms for each type of words in the present study. However, no greater priming effects of concreteness between concrete words and abstract words with respect to their corresponding translation equivalents in time window of 350–550 ms were found in forward translation and backward translation.

It has been found that N400 component was sensitive to semantic cognition load. As concreteness can be regarded as one part of semantic information, the N400 component is closely related to the priming effect of concreteness. The translation priming pairs elicited greater N400 than control pairs (non-translation priming pairs) in forward translation. One possible explanation is that the priming effects of concreteness in terms of N400 amplitudes come from the greater semantic overlap within translation pairs than within control pairs, which leads to the activation of more semantic features for Chinese primes than for English primes. Larger priming effects in forward translation than in backward translation in the time window of 350–550 ms for each type of words in the present study provided ERP evidence for the existence of translation asymmetry caused by the priming effects of concreteness.

However, no greater priming effects of concreteness between concrete words and abstract words with respect to their corresponding translation equivalents in time window of 350–550 ms were observed in two translation directions. This finding demonstrated that the priming effects of both concrete words and abstract words keep balanced in the two directions, and further suggested similar conceptual overlap between concrete words and abstract words with respect to their corresponding translation equivalents regardless of their concreteness. Indeed, the masked priming translation paradigm conducted in the present study is distinctive from the single lexical decision or semantic categorization task in which no context information was provided for the semantic knowledge of the target. More specifically, participants could only see the target without the primes in the single lexical decision or semantic categorization task. Concrete words would elicit greater semantic processing than abstract words (Barber et al., 2013). In the present study, it is assumed that compared with abstract primes, concrete primes may provide more specific semantic information for the targets to facilitate the semantic processing. However, both Chinese concrete primes and abstract primes offered quantitatively equal semantic clues to the English targets in L1–L2 translation direction, and both English concrete primes and abstract primes offered quantitatively equal semantic clues to the Chinese targets in the L2–L1 direction.

The balanced priming effects between concrete words and abstract words in ERP data analyses may be caused by SOA between primes and targets in the masked translation priming paradigm. Till now, it is still under debate whether or not concreteness of words can modulate the priming effects, since concreteness effects were SOA-sensitive, and only the priming paradigms within a certain range of SOAs could produce the facilitation effect of concreteness (Ferré et al., 2017). Chen et al. (2014) designed a study with 50 ms for the primes and 150 ms for the backward masks to investigate the concreteness effects in lexical decision task and semantic categorization task, and found no significant difference between concrete words and abstract words. Ferré et al. (2017) failed to find concreteness effects with the 50 ms SOA in the masked priming paradigm, but the concrete words showed greater advantages relative to abstract words in a 100 ms SOA. With the observed priming effects of concreteness in terms of N400 and the balanced concreteness effect in the 350–550 ms time window, we may have found the appropriate SOA for the studies of concreteness effect. In Chen et al. (2014) and Ferré et al. (2017), the primes lasting for 50 ms might not be so long enough to activate the targets, so the discrepancy between concrete words and abstract words disappeared in response latencies. The other possibility for the discrepancy of the related studies might ascribe to the technology. After all, ERP based studies are more sensitive to measuring the time course of processing, while behavioral studies mainly focus on the results of processing.

### The Role of Interplay Between Cross-Script Cognate Status and Concreteness

As discussed previously, it seemed that N150 is closely related to the processing of phonological information, while N400 is associated with concreteness. In the previous studies, the N250 component was thought to reflect the mapping of prelexical representations onto whole-word form representations (Holcomb and Grainger, 2006; Grainger and Holcomb, 2009). Chen et al. (2020) identified N300 component between P200 and P400 as the index of the morphological-semantic interface in Chinese–English non-cognates translation priming experiment. Therefore, it is possible that the P250 component elicited between N150 and N400 in the present study is closely related to the processing of phonological-semantic interface, and can be thought of as an index of phonological-semantic interface, reflecting the mapping of phonological information onto semantic representation.

For the P250 effect, the present study only found the main effect of word type in Experiment 2 (L2–L1 translation experiment), and no interaction effect between phonological similarity and concreteness effects was found in terms of P250 or in the time window of 200–350 ms. It seemed that the phonological and semantic features of English–Chinese cognates may not be closely related, and the phonological activation and concreteness representation were independent of each other regardless of translation directions in time window 200–350 ms, which may be explained by differences between English and Chinese. Unlike the close relationship between phonemes and meanings of phonography in English, Chinese characters are hieroglyphs and thus have relatively loose relation with the phonological features. Therefore, in Chinese–English cognate translation direction, no priming effects of the phonological-semantic interface were observed, but in English–Chinese cognate translation direction, P250 for main effect of word type was detected perhaps because more phonological-semantic overlapping information was activated in English cognates than in their Chinese equivalents. Therefore, it is crucial to further explore the interplay between cross-script cognate status and concreteness factors considering that lexical phonology is one of the routes accessing to semantic representation as illustrated in the BIA+ model.

## Conclusion

The present study investigated the concreteness effects of cross-script phonological activation with masked translation priming paradigm based on ERP technology in two experiments. N400 effect was found to be closely related to concreteness effects in Experiment 1. N150 and N400 effects were related to cross-script cognate effects and concreteness effects in Experiment 2. Greater priming effects of cross-script cognate status in cognates than in non-cognates for abstract words were found in the time window of 100–200 ms. Meanwhile, the translation asymmetry was observed in the time window of 100–200 ms with smaller priming effects for abstract cognates in forward translation than in backward translation, and in the time window of 350–550 ms with larger priming effects for each type of words in forward translation than in backward translation. We discussed the phonological activation and concreteness effects as well as translation asymmetry in view of the function of N150 and N400 components and the relevant models, mainly the Distributed Feature Model and Bilingual Interactive Activation (BIA+) model.

The present study only focused on the influence of cognate status and concreteness on bilingual memory, which cannot give a whole picture of bilingual memory. Additionally, we cannot deny that the development issues such as the age of acquisition may have great influence on the vocabulary learning of L1 and L2, and impact bilingual structure. Further cross-script studies might employ other techniques such as computational models to deal with as many variables as possible to examine phonological similarity and concreteness as computational models offer particular advantages in dealing with complex interactions between variables that are often confounded in natural language situations (Li and Zhao, 2018), which may shed more light on the principle of phonological activation and concreteness feature in bilingual visual word recognition.

## Data Availability Statement

The raw data supporting the conclusions of this article will be made available by the authors, without undue reservation.

## Ethics Statement

The study involving human participants were reviewed and approved by the Ethics Review Committee of College of Foreign Languages, Ocean University of China. The patients/participants provided their written informed consent to participate in this study. Written informed consent was obtained from the individual(s) for the publication of any potentially identifiable images or data included in this article.

## Author Contributions

SC, TF, and MZ put forward the idea of the study and contributed to the research design, experiments, and manuscript revision. YZ, YP, and LY contributed to the data collection, data analysis, and manuscript drafting. XG contributed to the research design, data analysis, and manuscript revision. All authors contributed to this article and approved the submitted version.

## Conflict of Interest

The authors declare that the research was conducted in the absence of any commercial or financial relationships that could be construed as a potential conflict of interest.

## Publisher’s Note

All claims expressed in this article are solely those of the authors and do not necessarily represent those of their affiliated organizations, or those of the publisher, the editors and the reviewers. Any product that may be evaluated in this article, or claim that may be made by its manufacturer, is not guaranteed or endorsed by the publisher.

## Footnotes

1
www.motif.org.au
